# A novel rotation method with variable-angle anterior probe for correcting the depth of the kidney to optimize renal dynamic imaging

**DOI:** 10.1186/s40658-022-00511-w

**Published:** 2022-11-17

**Authors:** Hua Wei, Ke Han, Jiaojiao Wu, Guang Hu, Zhifang Wu, Hongliang Wang, Xiaoshan Guo, Yansong Hou, Rui Wang, Sijin Li

**Affiliations:** 1 The Department of Nuclear Medicine, First Hospital of Shanxi Medical University, Taiyuan, China; 2 Beijing Novel Medical Equipment Ltd., Beijing, China

**Keywords:** Renal dynamic imaging, Renal depth, Rotation method

## Abstract

**Purpose:**

We aimed to investigate the effect and significance of the rotation method with variable-angle anterior probe corrected for the depth of two kidneys on the determination of glomerular filtration rate (GFR) in total and single kidneys by the renal dynamic imaging Gates method.

**Methods:**

Seventy-two patients who underwent dynamic renal imaging by the rotation method and abdominal CT in our hospital were collected in the present study. CT scanning, rotation method, Tonnesen's formula, and Li-Qian's formula were compared in terms of the depth of two kidneys, the depth difference between the two kidneys, and the total renal and single GFR obtained by substituting the renal depth values into Gates' formula.

**Results:**

**①**The depth of kidneys and GFR: Compared to CT, Tonnesen's formula significantly underestimated the depth of both kidneys and the total and single renal GFR (*P* < 0.05). No significant differences were found in the depth of both kidneys and the total and single renal GFR between Li-Qian's formula and the rotation method (*P* > 0.05), with a strong agreement and with the least bias in the values measured by the rotation method. ②Renal depth difference: Compared to CT, Tonnesen's formula and Li-Qian's formula underestimated the difference in depth between the two kidneys (*P* < 0.05). None of the differences were statistically significant based on the rotation method (*P* > 0.05). The depth difference was positively correlated with the resulting changes in single renal function (|R(CT)-R(Li-Qian)|) and (|R(Rotation)-R(Li-Qian)|) (*r* = 0.881, 0.641, *P* < 0.001). As the depth difference increased, Li-Qian's formula could not visualize changes in single renal function accurately. In contrast, the accuracy of the rotation method in assessing single renal function remains unaffected.

**Conclusion:**

The rotation method obtains an accurate depth and depth difference between the two kidneys without additional CT radiation, enhancing the accuracy of the Gates method for determining total and single renal GFR.

*Trial registration* Medical Ethics Committee of First Hospital of Shanxi Medical University, 2021BAL0146. Registered 12 January 2021.

**Supplementary Information:**

The online version contains supplementary material available at 10.1186/s40658-022-00511-w.

## Background

Glomerular filtration rate (GFR) is an excellent indicator of renal function, which is widely used for disease assessment and identification of complications after renal transplantation [1]. The renal dynamic imaging Gates method is a user-friendly, reliable, and efficient method to measure GFR. It is widely accepted among clinicians as it can quantitatively assess single renal function [2–4]. The precise estimation of renal depth helps to accurately determine total and single renal GFR by the Gates method [2].

Renal dynamic imaging Gates mostly uses the Tonnesen’s formula to estimate renal depth, which underestimates the actual depth [5, 6]. Qian Li et al. [7] measured renal depth in Chinese by CT scanning and derived a formula. To obtain GFR more accurately, Kun Li et al. [8] performed renal dynamic imaging of GFR by CT scanning to determine renal depth, but with the addition of extra radiation.

Considering the research background and current challenges, an innovative rotation method (Invented by Beijing Novel Medical Equipment Ltd.) was used to calculate the depth of kidneys of the subject by using a variable-angle dual-probe SPECT with an anterior probe for the multi-angle acquisition of projection data. We estimated the accuracy of the rotation method for calculating the kidney depth and the depth difference and evaluated the effect of the calibration of kidney depth on the determination of GFR by Gates renal dynamic imaging.

## Materials and methods

### Patients

This was a prospective study. It was approved by the Ethics Committee of the First Hospital of Shanxi Medical University, and all patients signed an informed consent form (clinical trial number: 2021BAL0146). Seventy-two patients (details in Table 1), including those who underwent renal dynamic imaging from January 2021 to December 2021 and abdominal CT scanning within one week before and after the imaging, were selected. There were 15 normal participants, 16 participants with mild hydronephrosis, 8 participants with renal agenesis (all with a diameter of < 2 cm), 19 participants with glomerulonephritis and diabetic nephropathy, and 14 participants with other problems (bladder tumor, prostatic hyperplasia, and ureteral stenosis with abnormal renal function). Patients with uremia, severe hydronephrosis, multiple cysts, and renal morphology disorders were excluded.

**Table 1:** General clinical data of the participants

Viable	Data
Gender (Male/Female, n)	37/35
Age ($\bar{\hbox{x}}\pm\hbox{s}$years old)	47.01 ± 16.23
Height ($\bar{\hbox{x}}\pm\hbox{s}$ m)	1.67 ± 0.09
Weight ($\bar{\hbox{x}}\pm\hbox{s}$ Kg)	66.71 ± 13.97
BMI ($\bar{\hbox{x}}\pm\hbox{s}$ kg/m^2^)	23.70 ± 3.48
Body surface area ($\bar{\hbox{x}}\pm\hbox{s}$ m^2^)	1.80 ± 0.23

### Renal dynamic imaging

**Fig. 1:**
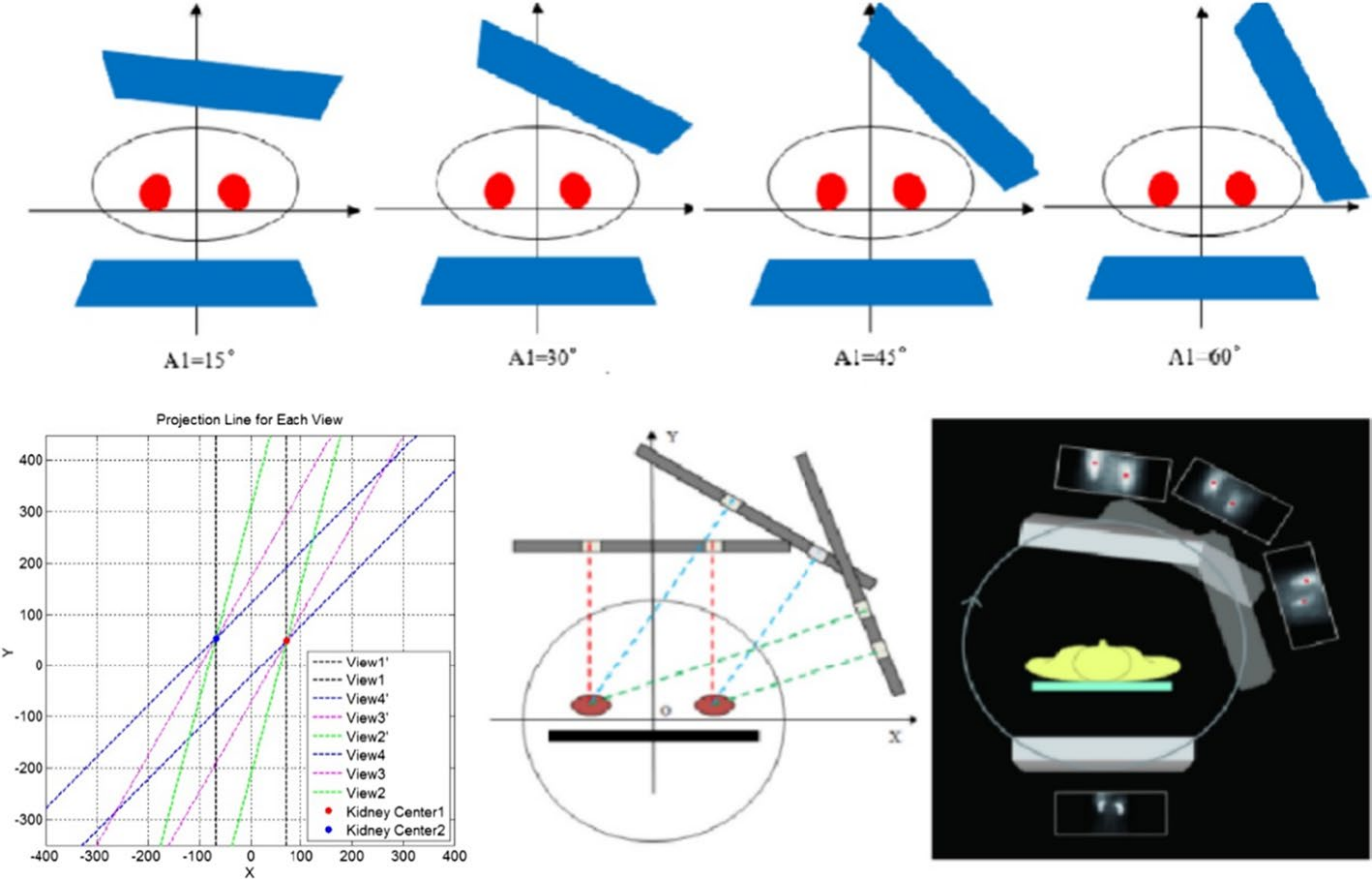
Schematic diagram of the rotation method. The process of acquiring the kidney projection image at different angles is shown. The projection image is segmented to obtain the center of gravity of the kidney projection at different sampling angles for calculating the spatial position of the center of gravity of the kidney projection, and then, calculating the projection line of the center of gravity of the kidney projection at different sampling angles based on the spatial position of the center of gravity of the kidney projection. The intersection of the projection line of the center of gravity of the kidney projection at all sampling angles is the actual center of gravity of the kidney

Imaging was performed using the NET632 dual-probe SPECT instrument from Beijing Novel Medical Equipment Ltd. The rotation method was developed by Beijing Novel Medical Equipment Ltd. in collaboration with our group to obtain the kidney depth and calculate GFR by collecting projection data from multiple angles with the anterior probe. The patient was placed in a supine position, the probe field included both kidney areas and the bladder, and the imaging agent ^99m^Tc-DTPA (^99m^Tc was provided by Atomic High Tech; DTPA was provided by Beijing Shihong Drug Development Center) was administrated by the “bolus” injection method into an elbow vein. The posterior images were acquired continuously for 20 min in dual time phases starting from the posterior probe, in which the renal artery perfusion phase was acquired at one frame every 2 s for 60 s and the renal function phase was acquired at one frame every 1 min for 19 min. The projection data were acquired by rotating the anterior probe at the third minute after the start of imaging, with four angles (15°, 30°, 45°, and 60°). Each angle was acquired for 2 min (Fig. 1). The collection conditions are as follows: low-energy high-resolution collimator (LEHR), energy peak 140 keV, window width 20%, and a matrix (64 × 64).

### Calculation of kidney depth methods

#### Rotation method

The principle of the method is tomosynthesis, which is to determine the target location by taking pictures in different directions. Here the posterior probe conducts traditional renal dynamic imaging; meanwhile, the anterior probe collects data from different angles. The anterior probe was rotated to collect projection data at 3 min after the start of renal dynamic imaging, and a total of four angles (15°, 30°, 45°, and 60°) were collected, with each angle collected for 2 min. The data acquisition procedure and kidney depth estimation method have been developed and integrated into the Human SPECT software by Novel Medical. The projection of the kidney center is approximately the center of the projection image. Segment the projection image of each angle, obtain the centroids of the segmented kidneys, and calculate the projection lines of the centroids by using the position parameters of the anterior probe for each angle, then the cross point of all the projection lines can be determined by optimized method, that is the real centroid of the kidney. The kidney depth can be calculated by the coordinate position of the kidney center and the bed (Fig. 1).

#### CT method

**Fig. 2:**
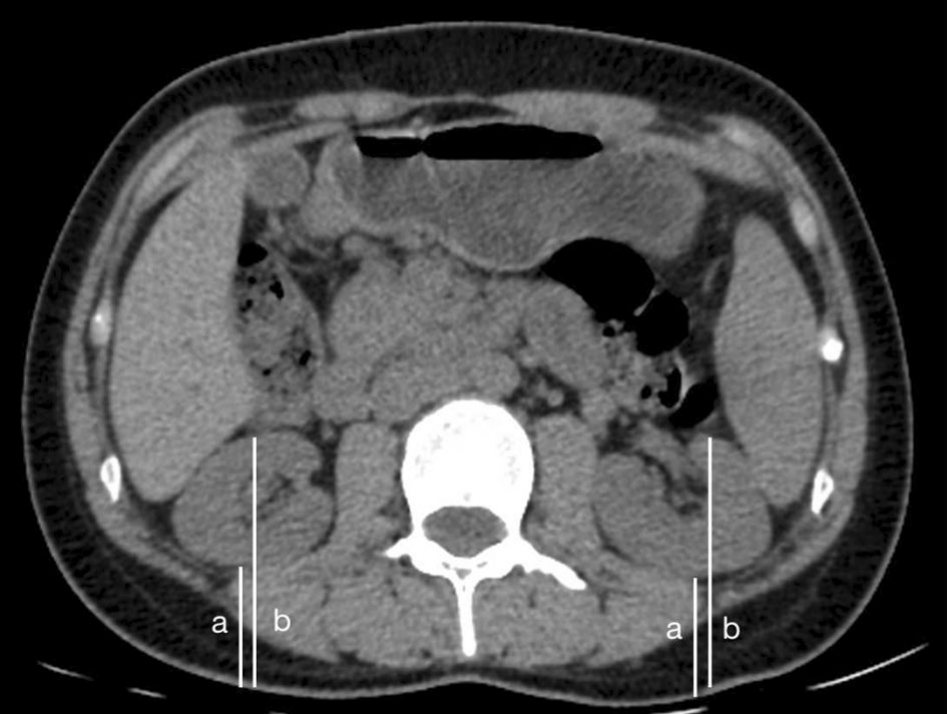
A CT image was taken to measure the renal depth. For posterior renal depth, renal depth (cm) = (a + b)/2

The horizontal CT imaging of the renal hilum was selected, and the vertical distances from the anterior and posterior edges of the kidney to the dorsal skin surface were measured separately; the average of the two was taken as the depth of the kidney. (Fig. 2).

#### Formula method

Tonnesen's formula was derived from ultrasound measurements of kidney depth in the white race [9].

$${\text{D}}_{{{\text{R}} }} =_{ } {13}.{3 } \times {\text{ W}}/{\text{H }} + \, 0.{7},{\text{ D}}_{{{\text{L}} }} =_{ } {13}.{2 } \times {\text{ W}}/{\text{H }} + \, 0.{7}$$

Li-Qian's formula was obtained based on the depth of the kidney measured by CT scanning in Chinese adults [7].

$${\text{D}}_{{\text{R}}} = { 15}.{449 } \times {\text{ W}}/{\text{H }} + \, 0.00{9637 } \times {\text{ Age }} + \, 0.{782},$$

$${\text{D}}_{{\text{L}}} = { 16}.{772 } \times {\text{ W}}/{\text{H }} + \, 0.0{1}0{25 } \times {\text{ Age }} + \, 0.{224}$$

Note that D_R_ is the right kidney depth, D_L_: left kidney depth; W: weight (kg), H: height (cm), and Age: age (years old).

### Image post-processing and GFR calculation

The self-contained software of the Novel Medical image processing workstation was applied. The patient's age, height, and weight were recorded, and the region of interest (ROI) of two kidneys and the background ROI were outlined on the functional phase image. The ROI was sketched by two nuclear medicine physicians, and the average of the GFR obtained by the Gates method was recorded by the rotation method, Tonnesen's and Li-Qian's formula. The CT-measured depth values of both kidneys were entered into the processing system to obtain the average of the GFR values obtained by the Gates formula after outlining the ROIs. Changes in single renal function were evaluated using the relative renal function, which refers to the ratio of single renal GFR to total renal GFR. The relative GFR values of the left (right) kidney measured by CT, rotation method, and Li-Qian’s formula were obtained from the Gates formula, and then the absolute value of the relative GFR difference was calculated: |R(CT)-R(Li-Qian)|, |R(rotation)-R(Li-Qian)|(R:Relative renal function (%) = GFR of left (right) kidney /total renal GFR).

## Statistical analysis

The SPSS 21.0 software was used for statistical analysis. Measures conforming to a normal distribution were expressed as the mean ± standard deviation (Mean ± SD), and the means between the two groups were compared by performing paired t tests. The Bland–Altman consistency test was performed to evaluate the degree of bias between the values measured by the rotation method, Li-Qian’s formula, and CT. The vertical coordinates of the Bland–Altman plot were M1-M2, and the horizontal coordinates were (M1 + M2)/2, where M1 was total and single renal GFR, the depth value or depth difference between the two kidneys measured by the CT, and M2 was by the rotation method or Li-Qian’s formula. The correlation between the depth difference between the two kidneys and the change in single renal function was analyzed by Pearson's correlation. The differences or correlations were considered to be statistically significant at *P* < 0.05.

## Results

Comparison of the depth, GFR and depth difference between the two kidneys, measured by the rotation method, Tonnesen's formula, and Li-Qian's formula with CT (Table 2).

**Table 2:** Renal depth, renal depth difference, and GFR values were determined by the four methods ($\bar{\hbox{x}}\pm\hbox{s}$)

Measurement method	GFR (mL/min)			Depth (cm)		Depth difference (cm)
	Total kidney	Left kidney	Right kidney	Left kidney	Right kidney	
Tonnesen's formula	64.76 ± 21.07^a^	30.70 ± 12.48^a^	34.06 ± 12.75^a^	5.94 ± 0.91^a^	5.98 ± 0.91^a^	0.04 ± 0.02^a^
Li-Qian formula	81.89 ± 25.57	38.93 ± 15.40	42.96 ± 15.58	7.34 ± 1.20	7.34 ± 1.11	0.07 ± 0.05^b^
Rotation method	81.68 ± 26.63	39.04 ± 16.00	42.64 ± 16.06	7.31 ± 1.26	7.29 ± 1.19	0.49 ± 0.37
CT	80.52 ± 25.98	38.60 ± 15.56	41.93 ± 15.41	7.29 ± 1.31	7.26 ± 1.19	0.39 ± 0.33

^a^: *P* < 0.001, Tonnesen's formula vs CT; ^b^: *P* < 0.001, Li Qian's formula vs CT

### The depth and GFR of two kidneys

The difference between kidney depth of Tonnesen’s formula and CT was statistically significant (left kidney: 5.94 ± 0.91 cm vs. 7.29 ± 1.31 cm, *P* < 0.001; right kidney: 5.98 ± 0.91 cm vs. 7.26 ± 1.19 cm, *P* < 0.001). The difference between Li-Qian’s formula and CT was not statistically significant (left kidney: *P* = 0.468; right kidney: *P* = 0.284). The difference was not statistically significant between the rotation method and CT (left kidney:*P* = 0.651; right kidney: *P* = 0.594). The Tonnesen’s formula significantly underestimated renal depth in both kidneys, and the Li-Qian’s formula and rotation method did not differ from CT.

The differences between GFR of Tonnesen's formula and CT were statistically significant (total kidney: 64.76 ± 21.07 ml/min vs. 80.52 ± 25.98 ml/min; left kidney: 30.70 ± 12.48 ml/min vs. 38.60 ± 15.56 ml/min; right kidney: 34.06 ± 12.75 ml/min vs. 41.93 ± 15.41 ml/min; *P* < 0.001). None of the differences were statistically significant between Li-Qian's formula and CT (all *P* > 0.05). None of the differences between the rotation method and CT were statistically significant (all *P* > 0.05). The Tonnesen’s formula significantly underestimated total and single renal GFR values, and the Li-Qian’s formula and rotation method did not differ from CT.

The depth differences between the two kidneys among the Tonnesen’s formula, Li-Qian’s formula and CT were statistically significant (Tonnesen’s formula: 0.04 ± 0.02 cm, Li-Qian’s formula: 0.07 ± 0.05 cm, CT: 0.39 ± 0.33 cm, *P* < 0.001), while the differences between the rotation method and CT were not statistically significant (rotation method: 0.49 ± 0.37 cm, CT: 0.39 ± 0.33 cm, *P* = 0.099). Both the Tonnesen’s and Li-Qian’s formulas significantly underestimated the difference in depth between the two kidneys, and there was no difference between the rotation method and CT.

### The degree of bias between CT and the rotation method, CT and the Li-Qian’ formula

The depth and GFR values of the two kidneys measured by the rotation method and the Li-Qian’s formula were generally closer to those measured by CT (Table 2). Therefore, the Bland–Altman method was used to compare the bias between the values measured by the rotation method and the Li-Qian’s formula and the values measured by CT.

**Fig. 3:**
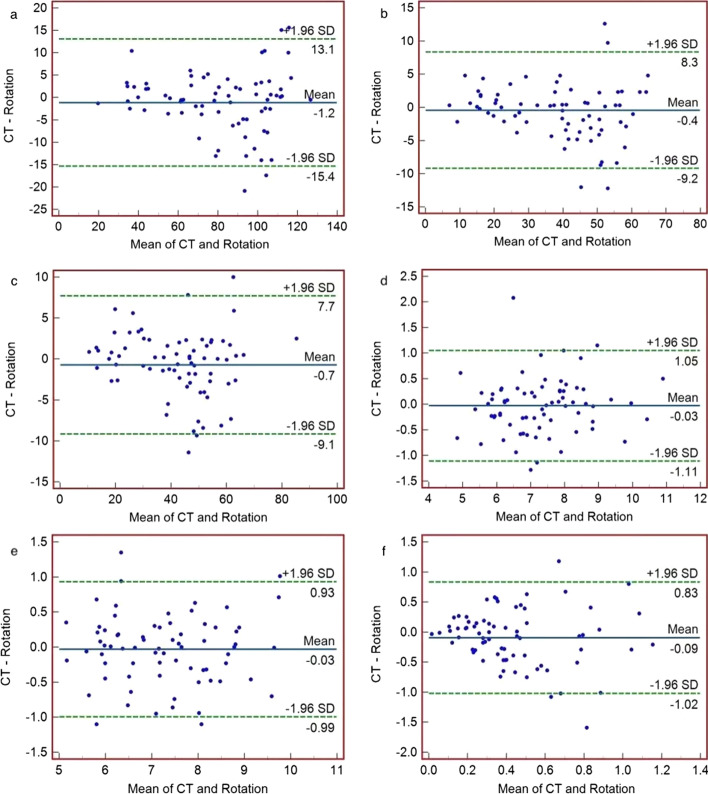
Bland–Altman plots of values were determined by CT and rotation methods in 72 patients. Note: In the figure, the three solid lines from top to bottom are the upper limit of agreement (LOA) of the difference (Xd + 1.96Sd), the mean of the difference (Xd), and the lower limit of the difference (LOA) (Xd-1.96Sd). Xd: mean of the difference; Sd: standard deviation of the difference.** a** total renal GFR values;** b** left renal GFR;** c** right renal GFR;** d** left renal depth;** e** right renal depth;** f** difference in depth between the two kidneys

**Fig. 4:**
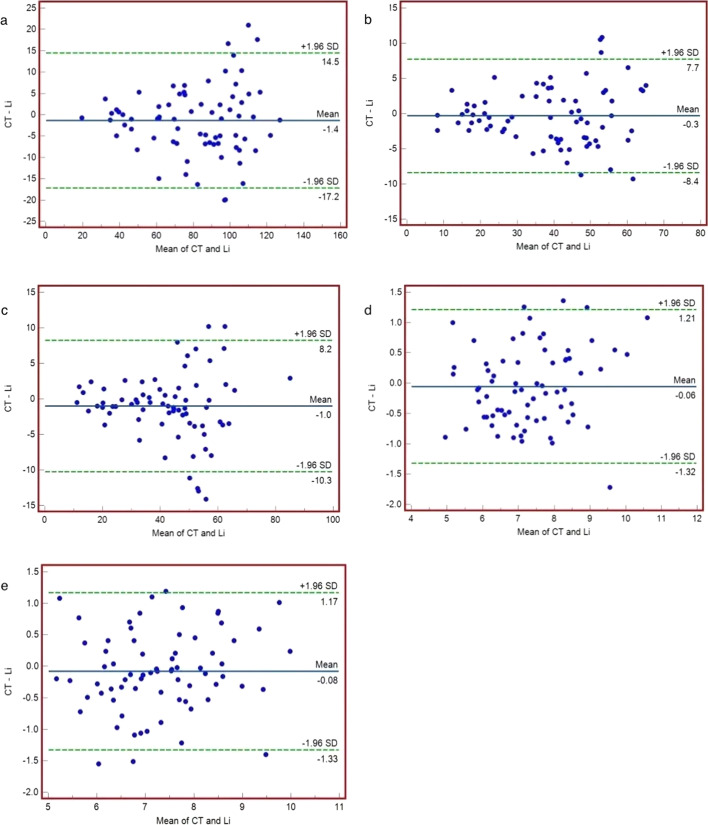
Bland–Altman plots of the values were determined by CT and the Li-Qian formula in 72 patients. Note: In the figure, the three solid lines from top to bottom are the upper limit of agreement (LOA) of the difference (Xd + 1.96Sd), the mean of the difference (Xd), and the lower limit of the difference (LOA) (Xd-1.96Sd). Xd: mean of the difference; Sd: standard deviation of the difference.** a** total renal GFR values;** b** left renal GFR;** c** right renal GFR;** d** left renal depth;** e** right renal depth;** f** difference in depth between the two kidneys

Rotation method compared to CT (Fig. 3), 5.56% (4/72) of the total and single renal GFR and left kidney depth points fell outside LoA, 6.94% (5/72) of the right kidney depth points fell outside LoA, and 4.17% (3/72) of the depth difference points fell outside the LoA. Li-Qian’s formula Compared to CT (Fig. 4), 6.94% (5/72) of the total renal GFR and left renal GFR points fell outside LoA, 6/72 (8.33%) of the right renal GFR points fell outside LoA, and 5.56% (4/72) of the left and right renal depth points fell outside LoA, respectively. The difference between the Li-Qian’s formula and the depth difference measured by CT was linearly correlated with the mean (*r* = 0.947, *P* < 0.001), and it did not meet the conditions for the application of Bland–Altman consistency analysis. Thus, no statistical tests were performed.

In conclusion, compared to CT, the rotation method and Li-Qian's formula yielded better consistency in total and single kidney GFR values and depth of both kidneys, and the difference in kidney depth measured by the rotation method was similar to that measured by CT.

### Depth difference and single renal function

The depth difference was 0.39 ± 0.33 cm with a range of 0.00 to 1.43 cm measured by CT and was 0.49 ± 0.37 cm with a range of 0.01 to 1.74 cm measured by the rotation method. The patients were divided into two groups according to the difference in depth (D) between the two kidneys measured by CT, D < 1.0 cm and D ≥ 1.0 cm, respectively. None of the differences between the values measured by the Li-Qian’s formula and CT were statistically significant. The renal depth difference and GFR measured by CT and rotation method are shown in Table 3.

**Table 3:** Renal depth difference and GFR values were determined by CT and the rotation method ($\bar{\hbox{x}}\pm\hbox{s}$)

Grouping	< 1.0 cm			≥ 1.0 cm		
	GFR (mL/min)			GFR (mL/min)		
	Left kidney	Right kidney	Frequency	Left kidney	Right kidney	Frequency
CT	39.18 ± 16.08	42.67 ± 16.09	61 (84.72%)	40.54 ± 19.67	42.23 ± 15.26	11 (15.28%)
Li-Qian formula	38.94 ± 15.26	42.70 ± 15.40		38.78 ± 18.38	45.89 ± 18.86	
*P* value	0.653	0.969		0.336	0.209	
Rotation method	38.72 ± 15.99	42.83 ± 16.01	62 (86.11%)	41.95 ± 17.06	40.88 ± 17.76	10 (13.89%)
Li-Qian formula	39.04 ± 15.51	43.37 ± 15.68		37.89 ± 15.44	39.14 ± 15.28	
P value	0.528	0.370		0.078	0.444	

**Fig. 5:**
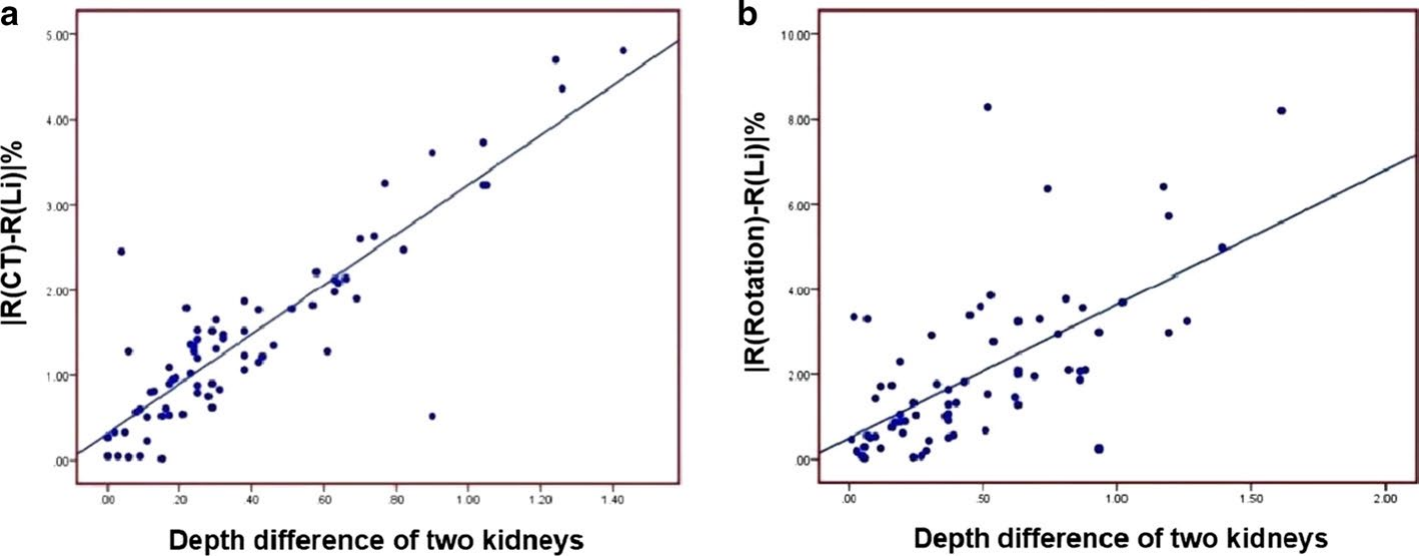
Changes in the depth difference between the two kidneys and single kidney function (**a**: CT—Li-Qian’s formula,** b**: rotation method—Li-Qian’s formula)

Pearson’s correlation analysis was performed to determine the correlation between the depth difference between the two kidneys and the changes in single renal function (Table 4). The results showed that the difference in depth between the two kidneys measured by CT was positively correlated with the difference in the left kidney (right kidney) relative renal function (|R(CT)-R(Li-Qian)|) between CT and Li-Qian’s formula (*r* = 0.881, *P* < 0.001) (Fig. 5a). The difference in depth between the two kidneys measured by the rotation method was positively correlated with the difference in the left (right) relative renal function (|R(rotation)-R(Li-Qian)|) between rotation and Li-Qian’s formula methods (*r* = 0.641, *P* < 0.001) (Fig. 5b). Thus, changes in the single renal function increased with an increase in the depth difference. The CT and rotation methods can reflect changes in single renal function with changes in depth difference, whereas the Li-Qian’s formula cannot accurately show changes in the single renal function in patients with large differences in depth between the two kidneys.

**Table 4:** GFR and the relative renal function were determined by the three methods

Measurement method	Left kidney (mL/min)		Right kidney (mL/min)		Absolute relative renal function difference (%)
	GFR	Relative renal function (%)	GFR	Relative renal function (%)	|R-R(Li-Qian)|
Li-Qian Formula	38.93 ± 15.40	47.42 ± 11.40	42.96 ± 15.58	52.58 ± 11.40	
Rotation method	39.04 ± 16.00	47.64 ± 11.92	42.64 ± 16.06	52.36 ± 11.92	2.03 ± 1.83
CT	38.60 ± 15.56	47.67 ± 11.18	41.93 ± 15.41	53.33 ± 11.18	1.46 ± 1.09

## Discussion

The renal dynamic imaging Gates method offers a reliable and noninvasive measurement of GFR [3, 4]. However, numerous elements can influence the determination of GFR by the Gates method [10]. The attenuation coefficient was 0.153 cm^–1^ in γ-rays in soft tissues, with a 14—16% deviation in GFR for every 1 cm change in kidney depth [11], making the correction for kidney depth particularly important.

In this study, a new rotation method was selected to use the multi-angle projection data of the anterior probe and calculate the depth of kidneys. It was more accurate than the formula method. It did not add any additional radiation dose. There was no significant difference between the renal depth measured by the rotation method and CT and GFR obtained by renal dynamic imaging.

### Renal depth measurement in multiple methods of GFR calculation

Tonnesen’s formula [6, 9] is the most commonly used formula for obtaining renal depth, with some limitations: only 55 white race subjects were included, and the age factor was discounted; ultrasound examinations were performed to measure the depth, and the position of kidney was different between ultrasound and renal dynamic imaging, which changed the position of kidney. And it is not applicable to Asians.

Several studies [5, 6] have shown that Tonnesen’s formula significantly underestimates the depth of kidney, which results in lower GFR. This study also confirmed these findings, with the Tonnesen’s formula significantly underestimating the depth and depth difference between the two kidneys and the total and single renal GFR.

The CT scan is the most accurate procedure for measuring renal depth [12], and Yang Yi et al. [13] demonstrated the feasibility of direct measurement by SPECT with CT instead of Tonnesen’s formula. The GFR measured by the ^99m^Tc-DTPA dual plasma method was recommended by the American Society of Nuclear Medicine Committee on Nephrology [14, 15]. Chen Li [16] considered the dual plasma method as the "gold standard" to evaluate the difference between the kidney depth measured by CT and Tonnesen's formula, respectively, showing a slight difference in the GFR between CT and the dual plasma method. However, CT adds additional ionizing radiation. Additionally, artifacts from respiratory motion can deteriorate the renal contours [17], so CT is not yet routinely used to measure renal depth.

The CT method is the reference standard used for establishing many formulas. Taylor [18] derived a new formula using CT measurements as the standard while incorporating age, but the study was based on the white race. Hui et al. [19] indicated that the Li-Qian’s formula was effective in improving the accuracy, but it failed to incorporate individual differences, and therefore, there were still unavoidable errors in the estimation of the depth difference between two kidneys.

In some studies, lateral plane images of the kidney were taken alone after completion of dynamic kidney acquisition [13, 20] to directly determine kidney depth, but this approach might not be suitable because peak uptake of the kidney usually occurs 2–4 min after injection of the developer, and the lateral images are not completed until after dynamic acquisition when intrarenal radioactivity is already at a low level, and body contours are blurred, compromising their measurement accuracy.

In this study, the rotational method was used, in which the anterior probe was rotated at 3 min after the start of renal dynamic imaging to acquire the renal projection images for a total of 8 min so that the whole acquisition process could be completed in the first 10 min when the renal image was clearly outlined. This allowed the measurement results to be highly accurate while allowing individual differences and without the need for additional acquisition time and additional radiation dose. In this study, the accuracy of the rotational method was also evaluated using the CT values as the standard, and the Tonnesen’s formula and the Li-Qian’s formula were compared. The results of the study showed no significant differences in total and single renal GFR and depth of the two kidneys compared to CT between the rotation method and Li-Qian’s formula.

### Renal depth difference of single renal GFR

Renal function was determined jointly by the left and right single kidneys. Single renal function measurement is an important component of the preoperative evaluation in many clinical contexts [21, 22]. The absolute single renal function includes absolute and relative single renal function [23]. The relative single renal function is the relative contribution of the left and right kidneys to total renal function, and the measures include the percentage of the relative function of the left and right kidneys.

From the formula of the Gates method [2], the single renal GFR can be obtained based on the ratio of the single kidney uptake rate to the dual kidney uptake rate: [(C_XK_-C_XB_)/e^–0.153YX^]/[(C_LK_-C_LB_)/e^–0.153YL^ + (C_RK_-C_RB_)/e^–0.153YR^]. The ratio of the single GFR value to the total GFR value is the relative renal function.

The difference in depth between the two kidneys affects the function of the single kidney. Gruenewald et al. [24] concluded if the two kidneys had the same renal function and the depth difference between the two kidneys was 1 cm, the single renal function ratio would change from 50:50 to 54:46. For a depth difference of 2 cm, it would change to 57:43, and for 3 cm, it would change to 61:39. Any formula would underestimate the depth difference between two kidneys. The depth difference between two kidneys estimated by Tonnesen’s formula was less than 0.1 cm for each subject, and that estimated by Li-Qian’s formula fluctuated within a small range.

In this study, the depth difference calculated by Tonnesen's and Li-Qian's formulas varied within a narrow range of 0.02 to 0.12 cm, whereas the depth difference between the two kidneys measured by CT was 0.39 ± 0.33 cm and by the rotation method was 0.49 ± 0.37 cm. Thus, both Tonnesen's and Li-Qian's formula significantly underestimated the depth difference between the two kidneys. The difference between the depth difference measured by CT and that measured by Li-Qian's formula and the mean value of the two were linearly correlated, which was probably because the depth difference measured by Li-Qian's formula was not accurately obtained and the depth difference measured was nearly constant.

In this study, although the differences between the two groups and the total and single kidney GFR values obtained from the Li-Qian’s formula were not statistically significant after grouping the kidney depths measured by CT and rotation, the depth difference was positively correlated with the resulting changes in single kidney function (|R(CT)-R(Li-Qian)|), (|R(Rotation)-R(Li-Qian)|) (*r* = 0.881, 0.641, *P* < 0.01).

Several scholars [18, 24] found the proportion of all subjects with a difference in depth between the two kidneys > 1 cm ranged from 7.5% to 42%. In this study, the proportion of patients with a difference in depth between the two kidneys ≥ 1 cm (measured by CT) was 15.28%. Thus, the changes in single kidney function due to differences in kidney depth also need attention. After grouping the kidney depths measured by CT and rotation method, the differences between the two groups and the total and single GFR obtained from the Li-Qian’s formula were not statistically significant. This might be because only a few cases were included in this study, and few cases had a difference in depth between the two kidneys ≥ 1 cm.

### Deficiencies

In this study, the exclusion of cases with severe hydronephrosis, and severe renal tumors to the point of renal displacement, might result in no difference in renal depth between CT, rotation method, and Li-Qian’s formula. For patients with severe renal failure or unilateral near-nonfunctioning kidney, the information of renal position cannot be identified due to low uptake of imaging agents, which in turn leads to the inability to accurately calculate the kidney depth. Since the anterior probe of the rotation method serves for positioning, it is not an option for patients with ectopic and transplanted kidneys. It is therefore limited in its scope of application which is a common problem with various methods. Furthermore, the rotation method involved in this study was based on the measurement of kidney depth to estimate GFR, and the correlation between poor kidney depth and the gold standard GFR needs to be confirmed.

## Conclusion

The Gates method incorporated a newly developed rotation method with variable-angle anterior probe which allowed simple, fast, and accurate estimation of the depth and depth difference between the two kidneys without additional radiation exposure to the subject, and a depth correction to the Gates method, which improved the accuracy of the Gates method for total and single renal GFR.

## Supplementary Information

**Additional file 1.** An article about the rotation method.

## Data Availability

The datasets used during the current study are available from the corresponding author upon reasonable request.

## Abbreviations

GFR: Glomerular filtration rate
SPECT: Single-photon emission computed tomography
CT: Computed tomography
ROI: Region of interest
