# Supplementary material for: A novel rotation method with variable-angle anterior probe for correcting the depth of the kidney to optimize renal dynamic imaging
Source: EJNMMI Phys. 2022 Nov 17;9:79. doi: 10.1186/s40658-022-00511-w (PMC9672276; doi:10.1186/s40658-022-00511-w)
Supplement: Supplementary file 1 — Additional file 1. An article about the rotation method. [file 40658_2022_511_MOESM1_ESM.pdf]

Simultaneous Kidney Dynamic Imaging and Depth Estimation with a Variable-angle Dual Camera SPECT

Lei Zhao, Si Chen
Beijing Novel Medical Equipment Ltd.
Beijing, China
zhaolei@novelmedical.cn

Daoyu Wang, Lilei Gao, Yaqiang Liu, Mai Liu
Beijing Novel Medical Equipment Ltd.
Beijing, China

***Abstract*—Kidney depth is an important factor affecting the estimation of glomerular filtration rate (GFR). Recently the most widely used method to estimate kidney depth in Radionuclide Dynamic Renal Imaging is Tonnesen formula. However Tonnesen formula has been proved to underestimate the kidney depth especially when the BMI of the patient is large or small. In this paper a method of simultaneous kidney dynamic imaging and depth estimation is proposed, based on the NET632 Single Photon Emission Computed Tomography (SPECT). The device, which is independently developed and produced by Novel Medical, has a variable-angle dual camera. The technology comes from tomosynthesis, which is to determine the target location by taking pictures in different directions. Here one camera conducts traditional renal dynamic imaging, meanwhile another collects data from different angles. Then the kidney depth is determined with image segment and some calculation of spatial location. The workflow and kidney depth estimation method have been integrated into the HumanSPECT software which is also independently developed by Novel Medical. Dynamic $^{99}Tc^{m}$-DTPA renal imaging was performed in 2 volunteers until now. The kidney depth estimated by Tonnesen formula and SPECT were compared with that measured by Computed Tomography (CT). Compared to the kidney depth measured by CT, the result of Tonnesen formula is significantly lower; the relative error goes up to more than 10% even 20%. As a result, the GFR indirectly calculated by Tonnesen formula is lower than that by CT. While the estimated kidney depth by SPECT is larger than CT measurement with relative errors below 10%, and the sequent GFR are in a reasonable range. The method by SPECT improves the accuracy of kidney depths as well as GFR.**

Keywords—Kidney depth; Glomerular filtration rate; Tonnesen formula; Tomosynthesis; NET632 SPECT; CT;

I. INTRODUCTION

The glomerular filtration rate (GFR) is traditionally considered the best overall index of renal function in health and disease (1). Currently, the widely adopted radionuclide renal dynamic imaging in clinical practice uses Gates' method to calculate GFR. However, the accuracy for estimation of GFR depends on a number of factors, including region of interest definition, background subtraction, linear attenuation coefficient of the radionuclide in the soft tissues, net injection dose, quality of “bolus-like” injection, kidney depth and so on.

Kidney depth is an important factor and variation in the skin-to-kidney center without correction for tissue attenuation has been shown to introduce errors in absolute quantification of kidney activity (2,3). Considering only the attenuation by soft tissue and assuming a linear attenuation coefficient of 0.153cm^{-1} for technetium-99m- ($^{99}Tc^{m}$) diethylenetriamine pentaacetic acid (DTPA), a 1-cm variation in organ depth will result in a 14-percent change in external measurements (4). Many correction methods for tissue attenuation are used by the investigators, like direct measurements of kidney depth using ultrasound or Computed Tomography (CT), formulas relating kidney depth to patient height and weight (5,6) such as Tonnesen formula, Taylor formula, Inoue formula, etc. However, many researchers have proven that the estimated kidney depth has a significant difference with the values measured by CT (7), especially when the patient is too thin or obese, also is not suitable for patients with renal transplantation or ectopic kidney.

NET632 SPECT, independently developed and produced by Novel Medical, is featured by two variable-angle cameras. Based on the device, a method of simultaneous kidney dynamic imaging and depth estimation is developed. Without extra procedure and time cost, relatively accurate kidney depths can be estimated at the same time of dynamic renal imaging.

II. METHOD

The principle of the method is tomosynthesis, which is to determine the target location by taking pictures in different directions.

On the transverse section of kidney hilum, kidney depth is the perpendicular distance from the dorsal skin surface to the kidney center. The projection of the kidney center is approximately the center of the projection image. Another advantage is during the first ten minutes, the radioactivity concentrates in the kidney with low background counts.

Traditionally dynamic renal imaging use only one camera, since NET632 SPECT has two cameras, one can be used to collect dynamic renal data, another can be used to collect data at different angles. The real kidney centers can be determined by calculating the cross point of the kidney center’s projection lines at different angles. Besides, the patient is on supine

position with his back contacting the bed surface closely; thereby the kidney depth can be determined by the coordinate position of the kidney centers and the bed. The data acquisition of the two cameras is independent and synchronous, which do not need extra procedure and time cost.

Some theoretical calculations are necessary to obtain proper position parameters of the camera at each angle. Proper position parameters can insure that kidney projections are entirely included in the useful view, and the room between the patient and the camera is suitable for patients of general body size, also make sure safe measurement without interference among patient, cameras and bed. Then, several dynamic renal imaging tests with kidney models are conducted to verify these position parameters and the data acquisition procedure.

A. *Data acquisition procedure*

Dynamic renal imaging was performed by using NET632 SPECT of Novel Medical. $^{99}Tc^{m}$- DTPA 5 mCi was the radionuclide used in the patient. The patient drank 500ml water before the test. A 6 seconds pre-injection count was performed by placing the syringe 30 cm from the center of the parallel-hole medium-energy collimator of the device. After the patient part of the study, a similar 6 seconds post-injection syringe count was performed. The radionuclide was rapidly injected intravenously into the supine patient who was prepositioned on the bed with ensuring that the kidneys were in the projection view. Matrix size of the renal data is 64*64, that of syringe count data is 256*256. Data acquisition was initiated at the moment of injection.

The camera below the bed collects dynamic renal data by two phases, the first phase of 30 frames with 2 sec of each frame, and then the second phase of 20 frames with 1 min of each frame. Meanwhile the upper camera moves to different positions at angles of 15, 30, 45, 60 degrees respectively, and collected data in a pre-set time slot. See Fig. 1.

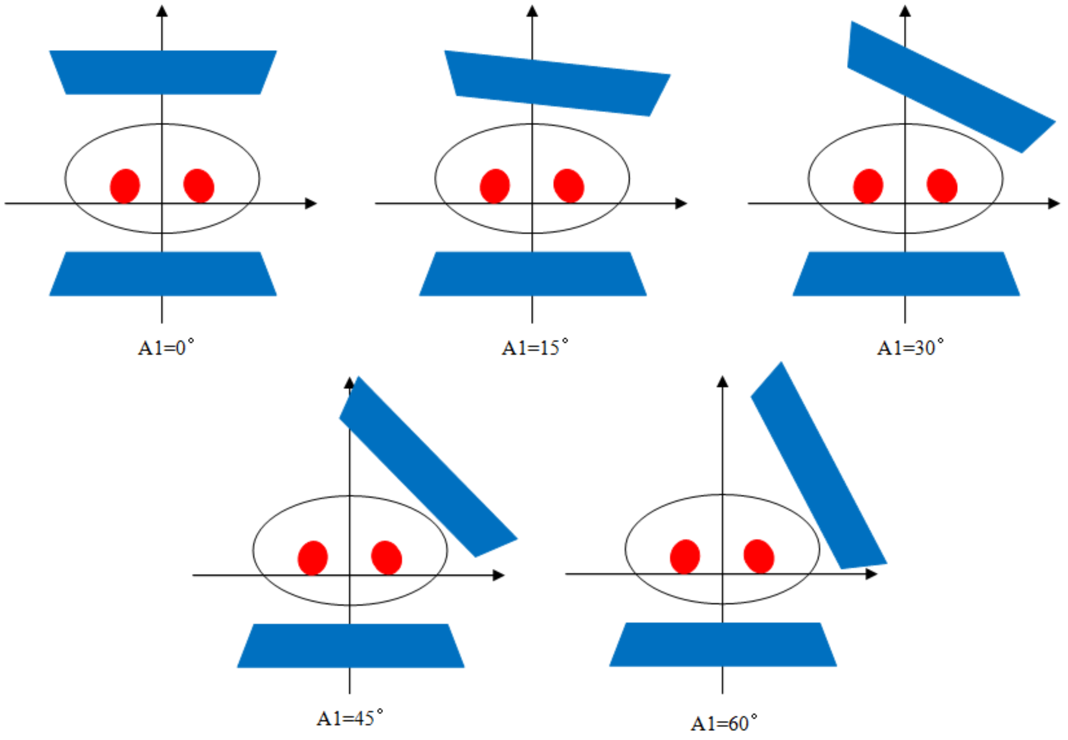

Fig. 1. Data acquisition procedure of NET632 SPECT

B. *Kidney depth estimation*

Perform image segment of the projection image of each angle, obtain the centroids of the segmented kidneys, and calculate the projection lines of the centroids of each angles by using the position parameters of the upper camera for each angle, then the cross point of all the angles' projection lines can be determined by optimization method, that is the real centroid of the kidney, see Fig. 2. The kidney depth can be calculated by the coordinate position of the kidney center and the bed.

Outline each kidney with its separate semilunar-shaped background region as an area of interest. The GFR estimation employs Gate's method. After the conclusion of process, the related renal parameters and the nephrogram are displayed.

The data acquisition procedure and kidney depth estimation method have been developed and integrated into the HumanSPECT software by Novel Medical. Under the options of "Kidney Depth Calculation", there is a new option of "Automatic computation by data of different angles" besides "Compute by Height and Weight" and "Manual Input".

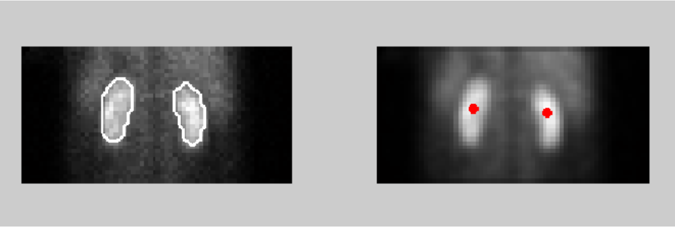

Fig. 2. Image segment and centroids calculation for kidneys.

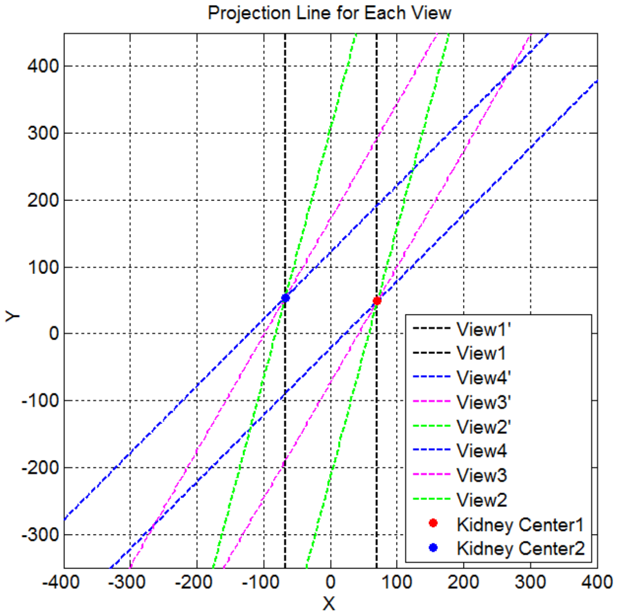

Fig. 3. Kidney centers determination by projection lines of all the angles.

III. RESULTS

This procedure and method has been performed on two volunteers, also CT of abdomen in the patients at Navy General Hospital were evaluated to determine kidney depths.

CT were performed on a GE PET/CT System, with the patient supine using a slice thickness of 3.8 mm. Kidney depth was determined by identifying the mid-transverse section of each kidney and measuring the perpendicular distance from the dorsal skin surface to the kidney center. Right and left kidneys were measured independently from the CT hardcopy images.

The two volunteers are all male and 35 years old. Height and weight are shown in the table. Kidney depths calculated by Tonnesen formula and SPECT were compared with the measured distances from skin-to-kidney center CT. Table 1, 2, 3 and 4 show the kidney depth results and relative error to the CT measurement results as well as GFR by Gate's method.

Compared to the kidney depth measured by CT, the result of Tonnesen formula is significantly lower; the relative error goes up to more than 10% even 20%. As a result, the GFR indirectly calculated by Tonnesen formula is lower than that by CT. While the estimated kidney depth by SPECT is larger than CT measurement with relative errors below 10%, and the sequent GFR are in a reasonable range. So compared to Tonnesen formula, the method by SPECT improves the accuracy of kidney depths and GFR estimation.

TABLE I. VOLUNTEER 1ST, LEFT KIDNEY

**Height (cm)**	**184**	**Estimation Method**		
**Weight (kg)**	**105**	***Tonnesen Formula***	***SPECT***	***CT***
Left Kidney Depth (cm)		8.23	9.97	9.43
Relative Error (%)		12.7	5.7	
Left Kidney GFR (ml/min)		40.42	53.46	49.06

TABLE II. VOLUNTEER 1ST, RIGHT KIDNEY

**Height (cm)**	**184**	**Estimation Method**		
**Weight (kg)**	**105**	***Tonnesen Formula***	***SPECT***	***CT***
Right Kidney Depth (cm)		8.29	10.12	9.95
Relative Error (%)		16.7	1.7	
Right Kidney GFR (ml/min)		35.01	44.98	43.68

TABLE III. VOLUNTEER 2ND, LEFT KIDNEY

**Height (cm)**	**170**	**Estimation Method**		
**Weight (kg)**	**70**	***Tonnesen Formula***	***SPECT***	***CT***
Left Kidney Depth (cm)		6.14	8.99	8.16
Relative Error (%)		24.8	10.2	
Left Kidney GFR (ml/min)		24.14	39.07	33.95

TABLE IV. VOLUNTEER 2ND, RIGHT KIDNEY

**Height (cm)**	**170**	**Estimation Method**		
**Weight (kg)**	**70**	***Tonnesen Formula***	***SPECT***	***CT***
Right Kidney Depth (cm)		6.18	9.05	8.34
Relative Error (%)		25.9	8.5	
Right Kidney GFR (ml/min)		29.10	47.20	36.23

IV. DISCUSSION

In the process of dynamic 99Tcm-DTPA renal scintigraphy by SPECT, soft tissues attenuation between the camera and kidney will result in counts decrease, so it is necessary to correct the counts by the kidney depth (skin-to-kidney center distances).

Recently, Tonnesen formula is still widely used in the GFR processing software. The formula is obtained with the patients sitting and the kidney depths are measured from lateral plain by ultrasonic imaging system. While SPECT scans the patient on supine position, postural change will affect the kidney depth. Therefore, Tonnesen formula will underestimate the kidney depth which will affect the tissue attenuation correction and then affect the accuracy of GFR. Based on the Gates formula, 10 mm deviation of kidney depth will lead to 14% deviation of GFR.

The method proposed in this paper, makes full use of the two cameras without extra time cost. The kidney depth estimated by SPECT is a little larger than CT measurement. Compared to Tonnesen method, the accuracy of GFR measurement by Gates method is improved by using kidney depths estimated by SPECT. In the near future, further studies are needed to verify the accuracy and stability of kidney depth estimation by SPECT.

An invention patent about the data acquisition procedure and kidney depth estimation method has been applied and is now in the stage of examination.

ACKNOWLEDGMENT

Supported in part by department of nuclear medicine, Navy General Hospital.

REFERENCES

[1] Smith HW. Diseases of the kidney and urinary tract. In: The Kidney: Structure and Function in Health and Disease. New York: Oxford Univ Pr; 1951:836-87.

[2] Kohn HD, Mostheck A. Value of additional lateral scans in renal scintigraphy. Eur J NucI Med 1979; 4:21-25.

[3] Hartling Oi, Marving J, Munck 0. Scintigraphy of kidneys located at different depths: the geometric mean method for determination of differential renal function. Clin Nucl Med 1987;12:956-957.

[4] Gruenewald SM, Fawdry RM. Kidney depth measurement and its influence on quantitation of function from gamma camera renography. Clin NuclMed 1985; 10:398-401.

[5] SchlegelJU, Hamway SA. Individual renal plasma flow de terminatiion2 minutesJ.Urol1976;116:282-285.

[6] Gates OF. Glomerular filtration rate: estimation from fractional renal accumulation of Tc-99m DTPA (Stannous).Am JRadiol 1982; 138:565-570.

[7] Taylor A, Lewis C, Giscometti A, et al. Improved formulas for the estimation of renal depth in aldults. J Nucl Med, 1993, 34: 1766-1769.
